# Participants' Comprehension of the Informed Consent in an Epidemiological Study on Dementia Prevalence: A Qualitative Study

**DOI:** 10.3389/fpsyt.2021.656822

**Published:** 2021-04-07

**Authors:** Ilaria Falvo, Maddalena Fiordelli, Rebecca Amati, Aliaa Ibnidris, Emiliano Albanese, Marta Fadda

**Affiliations:** Institute of Public Health, Faculty of Biomedical Sciences, Università della Svizzera italiana, Lugano, Switzerland

**Keywords:** informed consent, autonomy, ethics, epidemiolagy, dementia, qualitative study, Switzerland

## Abstract

**Aim:** In the absence of an effective treatment, informed participation in dementia research can hardly be underestimated. However, although informed consent is key in biomedical research, it may become a barrier to participation. Whether informed consent may cause confusion and contribute to unfair participant selection in dementia research is not known. In preparation of a future epidemiological study on the prevalence and impact of dementia in Switzerland, we aimed to conduct a qualitative study to explore participants' comprehension of the purpose of informed consent form and process shortly after participation in the pilot and validation study that preceded the large scale survey.

**Methods:** We conducted a qualitative study with 22 participants of the validation phase of an epidemiological study on the prevalence and impact of dementia in Switzerland to capture their understanding of both the nature and the content of the informed consent form and process. Participants were older adults (65 years or more) eligible for a dementia epidemiological study and their informant (a person who could provide information on their health and cognition). None of the participants reported to be suffering from dementia at the time of the interview.

**Results:** We found that participants held inaccurate and potentially trust-threatening beliefs regarding the scope of the informed consent. Participants identified contradictory contextual, formal and content needs that are difficult to be fulfilled, and misperceived the clinical and research settings in terms of informed consent procedures.

**Conclusions:** Participants and their proxies should be informed about both the scope of the informed consent process, and the content of the informed consent document in a focused, age-appropriate manner, while dispelling confusion about the purpose of research.

## Introduction

Contrary to mild cognitive impairment (MCI), the stage between the expected cognitive decline of normal aging and the more serious decline of dementia, dementia is a neurodegenerative syndrome characterized by progressive impairment in cognitive functions, including memory, reasoning, attention, and language (1). Dementia may be caused by different diseases and traumas primarily or secondarily affecting the brain, such as Alzheimer's disease or stroke (1). While consciousness may not be affected, dementia alters behavior and the ability to perform everyday activities (1). The most common form of dementia is Alzheimer's disease (60–70% of cases), a “primary degenerative cerebral disease of unknown etiology with characteristic neuropathological and neurochemical features” (1). Other major forms include vascular dementia, dementia with Lewy bodies, and a group of diseases that contribute to frontotemporal dementia (1). In the absence of an effective treatment, conducting both therapeutic and non-therapeutic research on dementia is crucial for prevention, and to reduce the burden on dementia on those who are affected, their family, and caregivers (2, 3). Dementia research is at the core of the seventh action area of the World Health Organization (WHO) Global action plan on the public health response to dementia (2), which highlights the importance of collecting up-to-date epidemiological data. The potential benefits of epidemiological research include obtaining new information about dementia etiology, diagnosis, and treatment, and about dementia costs, and the cost-effectiveness and use of healthcare (4). Researchers should promote and sustain high study participation rates among individuals both with and without dementia. Scarce participation can contribute to reduce both internal and external validity, thus limiting the generalizability of study findings (5). Factors that may contribute to older adults' exclusion from research include the complexity of the study design or ethical reasons (6–8), or to refusal to participate in research due to fear, concerns, or lack of trust (9–12). Moreover, evidence suggests that the informed consent process may also pose a potential barrier to participation in research among this age group (13, 14).

Informed consent is a cornerstone of research in human subjects (15) and, seeking to uphold the ethical values of participants' autonomy and their protection from harm, it represents one of its main requirements (16). However, studies have shown that the patient information sheet and declaration of consent might be a source of confusion among study participants. Participants may not understand the information presented during the informed consent procedure because the language is too complex, forms are excessively long, information is scant or of low quality, or the context where it is provided is not optimal (17–20). Three essential conditions must be fulfilled for informed consent to be valid, regardless of age: subjects should decide whether to participate freely without any coercion, they should receive sufficient clear, understandable, and usable information about the study, and be competent to understand such information and think rationally upon it (21). These conditions may be more difficult to meet at older ages (22). In particular, studies found that old age adversely affected recall of information offered in the informed consent form (23), and was associated to reduced understanding of informed consent information (24, 25). Only competent individuals can give informed consent for research; and even among them, it may be difficult or even impossible for those made vulnerable by sickness or dependency (26). Cognitive impairment may further limit the ability to actively participate in the process, even when consent is provided by a proxy or legal representative (27, 28). In many instances, the decision-making capacity is only partially impaired but declines during the course of a research project (13). Other factors, such as level of education and income, may introduce additional inherent vulnerabilities among eligible study participants (24, 29). Finally, older participants may not understand the purpose of the informed consent for cultural reasons (30).

In preparation of a future epidemiological study on the prevalence and impact of dementia in Switzerland, we aimed to conduct a qualitative study to explore participants' comprehension of the purpose of informed consent form and process shortly after participation in the pilot and validation study that preceded the large-scale survey. Specific objectives included exploring the meaning participants attributed to the informed consent process, their perceived barriers to accessing and understanding the informed consent form, and their preferences regarding the informed consent document's format and content.

## Materials and Methods

### Study Design

The present study constituted a qualitative follow-up investigation to a validation study conducted between March 2019 and October 2019 with 160 subjects in the Canton of Ticino, Switzerland. The validation study aimed at confirming the validity of the instruments to be employed in a soon-to-be study to assess the prevalence and impact of dementia in Switzerland. Inclusion criteria were being 65 or older for older adult participants, while there were no age restrictions for being an informant. All participants had to be resident in the Canton of Ticino, Switzerland. Each participant was also asked to identify a proxy, or “informant,”, i.e., a person who knew the participant well and could answer questions on his/her health. Of the 320 participants of the validation study (i.e., subjects and their informants), 35 agreed to be re-contacted for future studies and provided their personal contact information (e-mail address or telephone number). They were contacted by a member of the research team (RA) up to three times, who invited them to participate in an interview on their participation experience in the validation study. We informed prospective participants about the nature and scope of the present study and provided the necessary ethical safeguards (e.g., anonymity and confidentiality of the data, right to withdraw at any time, etc.). Recruitment lasted from December 2019 through January 2020. This article follows the COREQ (COnsolidated criteria for REporting Qualitative research) Checklist (31). The study was reviewed and approved by the Ethics Committee of the Canton of Ticino. The ethics committee waived the requirement of written informed consent for participation.

### Data Collection

Interviews were conducted in Italian between December 2019 and January 2020 by a member of the research team (RA, female, PhD) who has extensive experience in qualitative research and was employed as a postdoctoral researcher at the time of data collection. The interview setting was either the participant's home or the University building, according to participants' preferences. The interview resembled an in-depth conversation with open questions, where participants were invited to freely express their personal thoughts. Interview questions were developed *ad hoc* for this study, and elicited the meaning participants attributed to the informed consent process, their perceived barriers to accessing and understanding the information sheet and declaration of consent form, and their preferences regarding the document's format and content, including their opinion on using visual aids such as a video to support the informed consent process. Other questions elicited participants' motivation to participate in research and their preferences regarding the return of individual-specific and general study findings, which are the object of separate analysis. Interviews followed Holstein and Gubrium's “active interview” model, where interviewer and interviewee are conceptualized as equal and coactive in the production of knowledge (32). We adopted a flexible interview style, whereby participants were free to interrupt the interview whenever desired.

Each interview lasted approximately ~one 1 hour, was tape and video recorded (as materials would be later used to develop a campaign to boost participation rates in the epidemiological study), and transcribed verbatim. While one team member conducted and transcribed the interviews, a second team member double checked all transcriptions to guarantee a correct documentation of the data collected. Data collection was driven by data saturation, which happened when not novel insights could be extracted from the data. This condition was reached after 11 interviews. We collected data on participants' socio-demographics characteristics such as age, gender, occupation, and district of residence.

### Data Analysis

To identify the most significant and meaningful responses from the sample, two coders (IF and MF^*^) performed an inductive thematic analysis of the 19 transcripts based on Braun and Clarke's six-step approach (33). The two coders read all transcripts to familiarize themselves with the content, highlighting important quotes, identifying different labels, and organizing them in hierarchical order. Subsequently, we identified relationships between labels, highlighting thematic convergences and divergences. Discussion occurred between each stage of the analysis process and disagreement was resolved through discussion. Analysis of the transcripts was conducted in the original language (Italian) using NVivo12 by QSR software.

## Results

### Socio-Demographic Characteristics of Study Participants

The final sample included 22 individuals (11 women), including three couples who asked to be interviewed together. Those interviewed individually included 10 older adults who were eligible for the validation study as “participants,”, and 6 six informants who, at the time of the interview, were caring for a family member affected by dementia. One couple was composed of two friends, who were both older adults eligible for the validation study as participants. One was a couple of spouses, who were both older adults eligible for the validation study as both participants and informants (of each other). One was a couple of spouses composed of a participant and his informant. None of the participants reported to be suffering from dementia at the time of the interview. Among the informants, 2 two were daughters/sons and 7 seven were spouses of an actual dementia patient or an older adult eligible for the validation study. The average age was 71 years (SD = 9.3; range = 45 - −86). In terms of educational level, most participants either had completed high school (*n* = 10) or had a University degree (*n* = 8), were retired and resident in the Lugano district. See Table 1 for an overview of participants' socio-demographic characteristics.

**Table 1:** Study participants (*N* = 22).

**Variable**	***n***
**Gender of interviewees**	
Male	11
Female	11
**Age**	
45–64	4
65–69	6
70–74	4
75–79	4
80–84	3
85–89	1
**Nationality**	
European (non-Swiss)	4
Swiss	18
**District of residence**	
Bellinzona	4
Laventina	2
Locarno	2
Lugano	14
**Education**	
Primary	1
Secondary	11
University	9
**Occupation**	
Retired	17
Volunteer	5

### Themes Extracted From Participants' Reports

In general, participants identified a number of barriers to their comprehension of the informed consent form, including graphical and linguistic ones, and suggested ways to facilitate both the process of obtaining consent and their understanding of the informed consent document. The thematic analysis resulted in three themes related to the meaning attributed to the informed consent process. From the participant perspective, the informed consent process does not ensure full protection of study subjects. In addition, participants identified contradictory aspects. Finally, participants suggested that research and clinical informed consent is the same, they do not understand what research is.

#### Between Failed Protection and Trust

Participants' attached meaning to the informed consent process centered around two main positions. According to the first position, as the following participant reported, the informed consent form is a document is something to be looked at with suspicion, as it is mainly created to ensure protection of the study team, but not of participants.

“Clearly the person who wrote it, wrote it in favor of the person doing the research. He did not put himself in the participant's shoes… But this is the game of the parties” (Participant 2, older adult).

Participants reported to be aware that a PI may want to employ the informed consent form as a tool for protecting the study team from possible legal consequences.

“In certain situations, informed consent is just a formality… Something to download… But it is also understandable, now with all the lawsuits they receive in the hospital” (Participant 3, older adult).

A second position is defended by some participants who argued that the informed consent did not represent a barrier in their decision to consent to the study. They trusted the interviewer, the study team, and the University and, therefore, they also trusted the document. As two participants reported:

“Because I think you have no interest in cheating on people. […] I signed certain things. When the probability to be deceived is greater, much greater, then yes! But here, I went on trust” (Participant 17, older adult).

“There may be cases of people who may not understand and just sign because they trust you” (Participant 7, older adult and informant).

In particular, as the following participant explained, interpersonal contact with the interviewer or the study team replaced the function of the informed consent:

“Honestly, we went on trust because the lady called us on the phone, we heard her voice, she arrived here, we drank a coffee, we did all this…” (Participant 15, older adult).

#### Contradictory Needs That Are Challenging to Fulfill

The second thematic category explored the sample's perceived challenges related to accessing and understanding the informed consent. When we asked participants for their suggestions on how to improve both the informed consent process and the informed consent form, their reports were partly contradictory. Participants reported they would like to receive comprehensive information on the study, but at the same time they expressed their preference for a document which is as short as possible as they did not have enough time to read it. In particular, they reported that the informed consent form should always mention how anonymity, privacy, data protection, non-disclosure of data, information, and freedom to leave the study at any time are ensured.

“Those who participate want to be anonymized, indeed they demand it… This is the first point and on this you have to reassure them. The second point is the use of this information toward the participant himself, because in short it is like when I give my blood for testing or for other patients, I take it for granted that other people need it, I take for granted that it is examined, but I desire to stay anonymous. Respect, if unpleasant things are discovered, that I would like to be informed” (Participant 2, older adult).

“Giving the possibility and indicating at the end of the text that one can withdraw consent could be a guarantee and reassure those who participate” (Participant 18, older adult).

Participants also reported that the document should contain information on the study aim(s), study rationale, study process, and a section in which it is possible to provide consent to the return of individual-specific results. But, at the same time, this information should be provided in few words. For example, one participant stated:

“But if you could summarize it so that you could read it, it would be better… It should be summed up in four lines if you could […] You have to say in four words, in a schematic way, what the study aim is, how you will develop the results, if you want to have the results or not. These, I think, are the information which you have to provide” (Participant 5, informant).

They added that the form should be written in a simple language and important information should be placed on the first page so that participants can have access to it as soon as they start reading the document.

“Shorter is better, it is very simple. If it follows your rules, it becomes difficult, but the fact is: shorter is better” (Participant 17, older adult).

Participants emphasized that not enough time is devoted to read the informed consent form, partly because of lack of it and partly because people do not like reading.

“Look, if I read it, I did it as I am reading this, very quickly, one line every four to do it quickly” (Participant 7, older adult and informant).

“But I also know that people do not like reading. You can talk with any insurer… People refuse, they do not have time and then it is also difficult to understand” (Participant 2, older adult).

In addition, participants wished to obtain information as soon as possible to have time to read it at home, and at the same time they wanted to meet someone who provides the information. When asked when it would be the most appropriate moment to offer the informed consent form or its visual aids, all participants agreed on doing so as soon as possible. Participants suggested the possibility to have the document delivered at home before the first study meeting to have more time to read it carefully and reflect.

“When you have to sign something, you need to have time to read it and to think about it, so you could send it at home as soon as possible” (Participant 17, older adult).

“In my opinion, it would be more useful to send it a few days before the interview so that someone will read it at home and be able to say either yes, I do it, or I do not do it” (Participant 7, older adult and informant).

At the same time, participants reported that they wanted to be supported by the researcher during the informed consent process. Participants were aware of the time and resources required by this process, but they understood that the benefits that could derive would compensate for the investment. As two participants explained:

“I mean, you should have a person to explain what you are signing… Nobody did it and nobody does it because it is expensive and a waste of energy” (Participant 7, older adult and informant).

“I think that it is still important… Yes, the meeting to explain well. I know it is a matter of time, but you have to realize that time is also money, time is the best result” (Participant 15, older adult).

#### A Blurred Line Between Research and Clinical Settings

The interviewer clearly stated that the interview was about the informed consent process that happened as part of the validation study and not just any informed consent process that happens either in research or when we visit hospitals. Despite this, when we asked participants what they thought the scope of the informed consent was, a position embraced by many was that it is a mandatory document that necessarily needs to be signed in order to participate in research, similarly to situations in which they are asked to sign it in order to receive medical treatment. Participants reported to be aware of the fact that, if they failed to sign it, they would be excluded from the study, and perceived this instrument as a rigid, non-flexible tool to be accepted without any discussion or agreement.

“If I do not sign it, you do not do anything. I go home and the story is over… Right?” (Participant 18, older adult)

“No [I did not read it]. It is like when you go to the hospital, there is always this informed consent to be signed. It is a rule, it is mandatory.” (Participant 2, older adult).

As the following participant reported, the informed consent is seen as a customary procedure, something you “just have to do” and for which there is no alternative. Again, the participant made a parallel with the clinical setting.

“When you need to undergo a surgery at the hospital, they ask you sign something before [laughing]. I had to sign it for my brother in the hospital after he had a bike accident… And I said to myself: —Sign, which is the alternative? If I do not sign, you will do nothing…” (Participant 3, older adult).

Other highlighted that not enough time is allowed to patients to carefully go through the document before undergoing a medical procedure or treatment.

“I saw it in the hospital, you give it to the patient offhandedly and say: —Yes, yes, sign and go” (Participant 19, older adult).

## Discussion

The aim of this study was to explore, in view of an epidemiological study on dementia, participants' comprehension of the informed consent form and process. We explored the meaning participants attributed to the informed consent process, their perceived barriers to accessing and understanding the informed consent form, and their preferences regarding the informed consent document's format and content. We found that participants held inaccurate and potentially trust-threatening beliefs regarding the scope of the informed consent. Participants identified contradictory contextual, formal and content needs that are difficult to be fulfilled, and misperceived the clinical and research settings in terms of informed consent procedures.

A first finding is that participants were aware that the information sheet and declaration of consent have both legal and ethical foundations (34). Some argued that, from a legal point of view, their declaration of consent mainly serves the study team as a form of protection from possible lawsuits, while others viewed the informed consent process and document as a minor practice that is safeguarded by their trust in the study team and in the institution promoting the study. These findings expand our understanding of study participants' beliefs regarding informed consent. A previous investigation of the patient's awareness and understanding of the legal nature of informed consent in the clinical context found that 75% of patients falsely believed that it was a legal requirement (35). However, previous studies exploring participants' understanding of informed consent in the research setting started from the assumption that participants understand the intrinsic purpose of the informed consent process and rather focus on the comprehension of the different components of informed consent (36). The importance of discussing the ethical and legal role of the informed consent with participants has never been explicitly mentioned. Practices such as informed consent are meant to ensure the protection of future research subjects and their exercise of autonomy, but also to restore public trust in biomedical research (37). Lack of trust can severely endanger the whole biomedical research enterprise (38). Inaccurate beliefs regarding the purpose of informed consent may erode trust in investigators and research, and ultimately constitute a barrier to participation (39).

A second finding is that participants were aware that their understanding of study-related information and informed consent process may be impaired by lack of sufficient time, and graphic and content variables related to the information sheet and declaration of consent. For this reason, they reported a preference for a timely and short document and, at the same time, for comprehensive and interpersonal explanations regarding the study. These needs are difficult to meet simultaneously. This finding is echoed by previous evidence showing that elderly participants may require more time to mentally process information than do younger participants (21). Studies also found that elderly participants consenting to study participation needed more time to make a decision compared to those who decided not to participate (40). Impaired eyesight and visual acuity that accompany the aging process may influence the subject's ability to perceive information in a written form (27, 41). Studies showed that years of education and level of reading might also affect older participants' ability to comprehend the content of informed consent forms (19, 25, 27). However, the fact that our sample was highly educated partly contradicts this finding, suggesting that even participants with many years of education may need simplified informed consent forms and interpersonal support. Several studies have been conducted to improve participant understanding of the informed consent, but mixed evidence is available on their effectiveness (42). For example, the effectiveness of providing shorter informed consent form or using multimedia on improving participants' understanding is still questioned (43–46). Systematic comparisons of the literature found that enhanced consent forms (with increased readability) and extended person-to-person interactions and discussions were the two most effective strategies in improving participant understanding (44, 47). In light of our study results, the success of the latter strategy does not surprise. Our participants recognized that a committed and supportive presence of the investigator is an important element that facilitates a truly informed consent (34), suggesting that the manner and context in which information is conveyed is as important as the information itself.

A third finding was that participants viewed the informed consent process as a customary procedure for which there is no alternative, highlighting a conceptual blurring of the line between the research and medical/clinical treatment contexts. In this sense, they reported to feel compelled to sign the informed consent for the validation study without questions, in line with their experience in the medical care setting. Concerns about the boundaries between research and standard clinical care are not new. For the past forty 40 years, bioethics scholarship and research ethics guidelines have argued that informed consent to participate in research should include clarification of the differences between these two activities (48–54). In line with our findings, previous studies have found that some research participants do not appreciate important differences between clinical research and treatment, a phenomenon called “therapeutic misconception” (TM) (55, 56). Study participants who are unaware of study design implications, especially random assignment to a control or comparison group, may believe that they are assigned a medication based on what is best for them, personally. Not adequately appreciating the purpose and methods of research studies might compromise these participants' ability to evaluate risks and benefits of study participation (57). Our study results expand the evidence on the phenomenon of TM in psychiatric research (58), suggesting that such misconception may not only occur in the clinical research context but also in epidemiological study settings.

Our results have a number of practical implications. Our study reiterates that presenting study information in a disorganized and rapid fashion, allowing too little time for consideration or curtailing opportunities for questioning, all may adversely affect a subject's ability to make an informed choice and ultimately question the validity of the informed consent procedure among older participants (50). As other initiatives have suggested, it is of paramount importance to define an effective informed consent process, train research staff on best practice to inform prospective study participants and obtain consent, and improve the informed consent document (51). This should be presented as a tool first and foremost aiming at protecting their health. Participants should also be informed that, for an ethics committee, approval of an informed consent is mandatory.

### Limitations

Some limitations of this study are worth noting. First, we cannot exclude possible selection bias because participants had already taken part in a previous validation study. Their positive attitude and proneness to research participation may be related to the opinions expressed about the consent form and process. However, this might have given them a chance to reflect upon the informed consent before being interviewed, enriching their reports during the interview. Second, due to the face-to-face nature of the interview and the presence of a video-maker, social desirability bias may also have occurred. Nonetheless, the interviews setting was informal, and the interviewer adopted a nonjudgmental approach during the interview, and we offered participants to choose their preferred place to be interviewed. Third, many of the answers of the participants involved ““trust”” as a significant element of the informed consent process. In this sense, there are two variations of the interview setting that may have influenced participants' trust in the interviewer: (1) the interview being conducted alone vs. with a partner, i.e., a person of trust (in this case, the presence of participant's partner might facilitate the relationship with the interviewer); (2) interview conducted at the University vs. at home (in this case, a familiar, trustful environment might facilitate the establishment of rapport with the interviewer). While the variation of the interview setting might have influenced the interview results, we always ensured that both interviews conducted alone and at the University took place in a warm, non-judgmental environment.

International guidelines suggest that, for responsible epidemiologic research practice to take place, participants should be well- informed about the study and what is asked from them, and they should all sign an informed consent form before any study-related procedures are initiated (59, 60). As potential participants of epidemiological studies into dementia are likely to be part of vulnerable groups (due to their older age, possible cognitive decline, and presence of co-morbidities) (13), it is crucial that this document is prepared with care, using methods developed in consultation with them and their proxies, and taking into account their beliefs, needs and capacities (12). This can be best done by combining both quantitative and qualitative research approaches when engaging potential participants and their proxies. For example, investigators may ask for a stepwise consent procedure, where comprehension, risk and inconvenience scores can be obtained before and after the study procedures by asking closed-ended questions about the study's essentials (61). Other proven methods include the use of large print and simplified language, a storybook, and a videotape (62). While providing information to participants and their proxies on the study's main elements, investigators should also clarify and disclose the scope of the informed consent process, including its ethical and legal foundations. This should be done in a focused, locally appropriate manner and within a continuous informed-consent framework, ensuring application of the best competency assessment instruments and dispelling confusion about the scope of research (63, 64). This is likely to result in high levels of comprehension, information retention and, ultimately, and participation rate.

To prevent exploitation of human subjects and build true collaborative research partnerships with prospective and actual participants, researchers conducting epidemiological research must consider the plethora of ethical challenges posed by the informed consent process and document for older participants. Failing to do so will result in this instrument becoming a source of discrimination and an obstacle to not only participation but to the real exercise of participants' autonomy, which this tool is indeed designed to protect and sustain.

## Data Availability Statement

The datasets generated during and/or analyzed during the current study are not publicly available due privacy reasons but data summaries are available from the corresponding author on reasonable request. Requests to access the datasets should be directed to Marta Fadda (marta.fadda@usi.ch).

## Ethics Statement

The study was reviewed and approved by Comitato Etico del Canton Ticino. The ethics committee waived the requirement of written informed consent for participation.

## Author Contributions

RA made all the contacts with the participants and conducted the interviews. MF and IF led the analytic process and analyzed the data. RA, MF, and EA contributed to the study design. MF verified the findings of the analysis. All authors contributed to writing the paper.

## Conflict of Interest

The authors declare that the research was conducted in the absence of any commercial or financial relationships that could be construed as a potential conflict of interest.

## Supplementary Material

Supplementary Material 1 Interview grid.
