# Supplementary material for: Participants' Comprehension of the Informed Consent in an Epidemiological Study on Dementia Prevalence: A Qualitative Study
Source: Front Psychiatry. 2021 Apr 7;12:656822. doi: 10.3389/fpsyt.2021.656822 (PMC8058191; doi:10.3389/fpsyt.2021.656822)
Supplement: Supplementary Material 1 — Interview grid. [file Table_1.DOCX]

Appendix 1. Interview grid

| Participation to the validation study | |
| --- | --- |
| Participation experience | How was your experience of participating in the validation study?  What did it mean for you to participate in the validation study?  What do you remember from participating? What was the purpose of the study?  How did you feel during participation?  After participating in the validation study, what expectations did you have concerning the next phases of the project? |
| Motivation to participate | What motivated you to participate in the validation study?  Why do you think other people participate?  Why do you think other people do not participate?  Would you participate if you received an invitation to participate in an epidemiological study on the prevalence of dementia in Switzerland?  [If participant answers “yes” to the previous question] Let us pretend I am one of your friends. Unlike you, I do not want to participate. What would you tell me to convince me? |
| Barriers to participation | Is there anything or someone who made you hesitate with respect to participation?  Was there anything or anyone who pushed you to participate?  In your opinion, what should we do to ensure a high response rate to the epidemiological study? |
| Return of study results | |
| General opinion on the return of study results | Overall, what do you think about the fact that researchers communicate the study results to participants?  What do you think of the fact that researchers report the results of the tests you have been administered? |
| Understanding of the type(s) of study results | What types of results would you be interested to know? |
| Preferences regarding the communication of study results | How would you like the results to be communicated to you, if you agreed to receive them (in writing, verbally)? |
| Preferences regarding who to involve in the communication of the study results | Whom would you want to be informed of your results, if you agreed on their return? |
| Preferences regarding when to communicate the study results | When would you like to be informed of the results, if you agreed on their return? |
| Feelings associated with the return of study results | What feelings come to your mind if you think about having the results communicated to you? |
| Informed consent | |
| General opinion on informed consent | When you participated in the validation study, do you remember if you signed a document?  If so, what do you remember regarding the document you signed?  In general, what do you think about the fact that study participants give their consent to the return of study results? |
| Preferences regarding informed consent procedures | How would you like to provide your consent regarding the return of study results? |
| Preferences regarding who to involve in the informed consent form | Think about the individuals you want to be informed of your study results. Would you include these individuals in the consent? |
| Preferences regarding when to provide informed consent on the return of study results | When would you like to give your consent regarding the communication of the results? |
| Meaning of informed consent process | What do you think informed consent is meant for? What is its scope? |
| Perceived barriers | Was there anything that prevented you from understanding the information sheet and declaration of consent form? |
| Preferences regarding the document’s format and content | Is there anything you would change regarding the document’s format and content? What do you think about using visual aids such as a video to support the informed consent process? |
| Other questions |  |
| Dementia-related challenges | In your opinion, what are the biggest challenges in relation to dementia and Alzheimer's disease?  What and whom should researchers invest on?  What do you expect from research, in general? |
